# Laparoscopically Resected Foregut Cyst Adjacent to the Right Adrenal Gland

**DOI:** 10.1155/DTE.5.53

**Published:** 1998

**Authors:** E. Yamamoto, H. Nakayama, N. Ozaki, Y. Kitamura, M. Funatsuka, M. Ueda, K. Chikugo, A. Hirata, A. Kurashina, H. Kuratsuka, M. Nakagawa, S. Nagaoka

**Affiliations:** 1 Department of Surgery Shimane Prefectural Central Hospital 116 Imaichi-cho Shimane Izumo 693-8555 Japan; 2 Department of Pathology Shimane Prefectural Central Hospital 116 Imaichi-cho Shimane Izumo 693-8555 Japan

## Abstract

A case of 49-year-old woman with a retroperitoneal undifferentiated foregut cyst attached to the right adrenal gland is reported. The bronchogenic cyst is a type of foregut cyst with a cartilage component, but in this case the multicystic tumor lacked both cartilage and gland. It is quite rare among retroperitoneal tumors and has not been reported so far to have malignant potential. The preoperative diagnosis was an adrenal benign incidentaloma, and the patient successfully underwent laparoscopic resection of the cystic tumor together with the right adrenal gland by lateral transabdominal approach. Laparoscopic surgery for a retroperitoneal tumor is problematic, however, since benignancy cannot be predicted. In laparoscopic adrenalectomy for non-functioning adrenal tumor, therefore, a differential diagnosis from retroperitoneal tumor should be given serious consideration.


*Diagnostic and Therapeutic Endoscopy*, Vol. 5, pp. 53–56
Reprints available directly from the publisher
Photocopying permitted by license only

© 1998 OPA (Overseas Publishers Association) N.V.
Published by license under
the Harwood Academic Publishers imprint,
part of The Gordon and Breach Publishing Group.
Printed in Singapore.

### Laparoscopically Resected Foregut Cyst Adjacent to the Right Adrenal Gland

E. YAMAMOTO[a,*], H. NAKAYAMA[a], N. OZAKI[a], Y. KITAMURA[a], M. FUNATSUKA[a], M. UEDA[a], K. CHIKUGO[a], A. HIRATA[a], A. KURASHINA[a], H. KURATSUKA[a], M. NAKAGAWA[a] and S. NAGAOKA[b]

[a]*Department of Surgery,* [b]*Department of Pathology, Shimane Prefectural Central Hospital, 116 Imaichi-cho, Izumo, Shimane 693-8555, Japan*

*(Received 3 March 1998; In final form 30 March 1998)*

**A case of 49-year-old woman with a retroperitoneal undifferentiated foregut cyst attached to the right adrenal gland is reported. The bronchogenic cyst is a type of foregut cyst with a cartilage component, but in this case the multicystic tumor lacked both cartilage and gland. It is quite rare among retroperitoneal tumors and has not been reported so far to have malignant potential. The preoperative diagnosis was an adrenal benign incidentaloma, and the patient successfully underwent laparoscopic resection of the cystic tumor together with the right adrenal gland by lateral transabdominal approach. Laparoscopic surgery for a retroperitoneal tumor is problematic, however, since benignancy cannot be predicted. In laparoscopic adrenalectomy for non-functioning adrenal tumor, therefore, a differential diagnosis from retroperitoneal tumor should be given serious consideration.**

*Keywords:* Bronchogenic cyst, Endosurgery, Foregut cyst, Laparoscopic adrenalectomy, Retroperitoneum

### INTRODUCTION

Adrenal tumors diagnosed as benign rather than malignant prior to operation have recently been resected with laparoscope. A rare case of a retroperitoneal tumor presenting itself as an adrenal gland-origin mass was treated laparoscopically. The final diagnosis was an undifferentiated foregut cyst.

### CASE REPORT

A 49-year-old Japanese woman consulted a gastroenterologist at our hospital for further examination of a right adrenal swelling detected by screening ultrasonic study (Fig. 1). Computed tomography revealed a $32 \times 22$ mm tumor with clear boundary at the retroperitoneal paravertebral region superior to the right kidney (Fig. 2(a)).

* Corresponding author. Tel.: 0853 22 5111. Fax: 0853 21 2975.

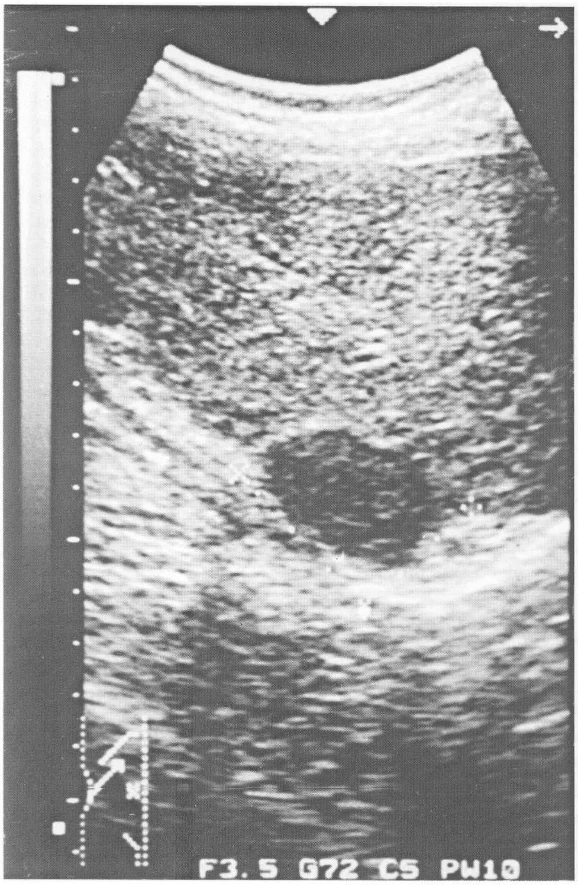

FIGURE 1 Abdominal US. A hypoechoic homogeneous mass was found posterior to the liver.

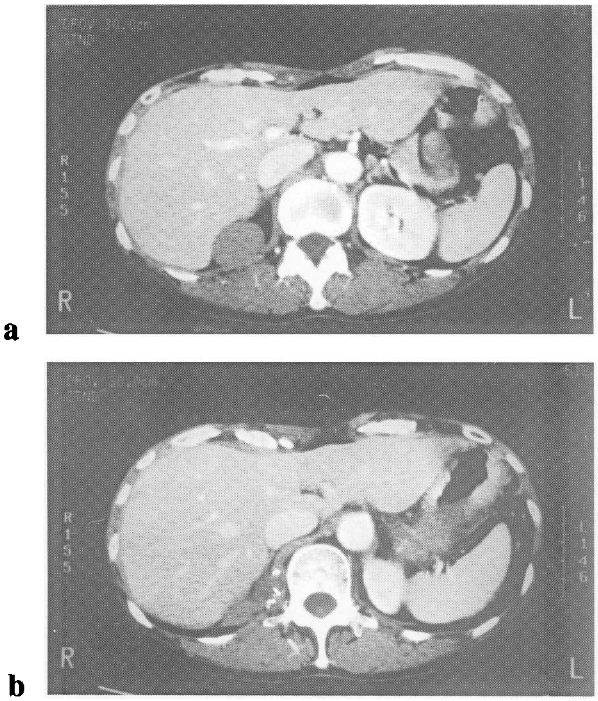

FIGURE 2 Abdominal CT scan. CT scan detected cephalad to the right kidney a tumor which was in part oval-shaped with soft tissue density (a) and which in another part was irregular-shaped and calcified (b).

The tumor was comprised of soft tissue with scattered calcification at its cephalad edge (Fig. 2(b)). She was referred to a endocrinologist and subjected to thorough hormonal examination, with the resulting diagnosis of non-functioning adrenal tumor. The incidentaloma of the adrenal gland 3 cm or larger in diameter is considered indication for resection, and so she was admitted to our surgical ward. At the operation theater, under general anesthesia, she was put in a left lateral decubitus position (Fig. 3), and three trocars were inserted into the peritoneal cavity. The right hepatic triangle ligament was incised and the liver was detached from the lateral abdominal wall, where it shifted down outside of view by its own weight, allowing the suprarenal region to be visualized clearly. A fourth port was made and an Endo

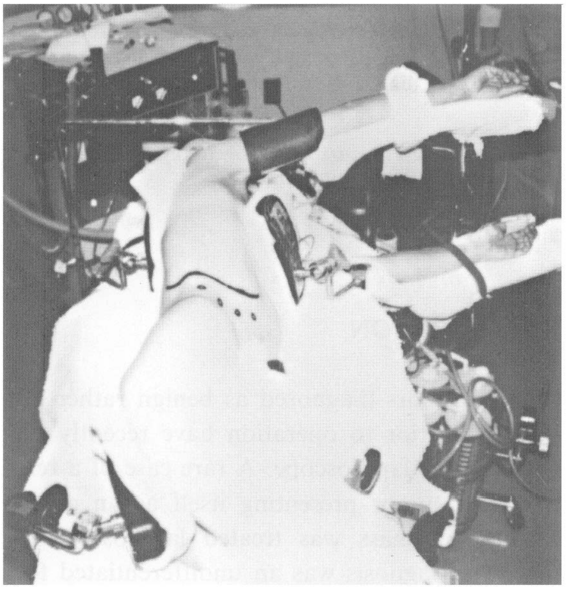

FIGURE 3 Operative position and trocar-sites. The patient was put in left lateral decubitus position, and padded at chest and pelvis so that the table could be rotated. Ports were made 5 cm apart from the costal margin at almost regular intervals from the mid-clavicular line to the mid-axillary line.

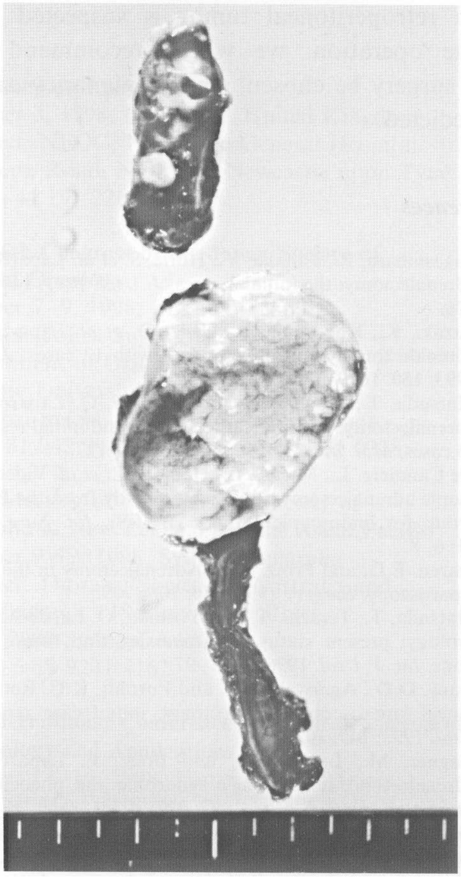

FIGURE 4 Formalin-fixed specimen. The tumor consisted of two parts; one was solely cystic containing very viscous fluid and the other was multilocular with wall thickening.

Paddle Retract (Auto Suture, Japan Inc.) was inserted in order to lift the liver and adjust the counteraction appropriately with minimal labor. The retroperitoneal serosa was incised and Gerota's fascia around the adrenal gland was dissected by laparosonic coagulating shears (Harmonic Scalpel, Ethicon Endo-Surgery). The tumor was located adjacent to the superior end of the adrenal gland, and was dumbbell-shaped. The narrow ligament connecting the two parts of the tumor was clipped and incised, and the inferior part was removed together with the adrenal gland. The superior part was isolated separately and removed through a trocar wound using Endo Pouch II (Ethicon Endo-Surgery). The tumor was found to be cystic (Fig. 4) and contained brown

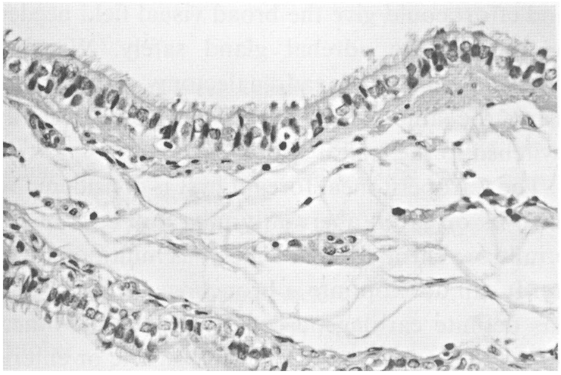

FIGURE 5 Microphoto of tumor. The cyst was lined with ciliated epithelium without any glandular nor cartilageous differentiation.

mucus of high viscosity. The operation was successful, and the patient was discharged a week later uneventfully and soon returned to work. Pathohistological study revealed that the cyst was lined with ciliated epithelium (Fig. 5), and had underlying smooth muscle in another portion, but lacked cartilage or gland, leading to a final diagnosis of undifferentiated foregut cyst.

### DISCUSSION

Laparoscopic adrenalectomy was conceived by Japanese urologists in 1992 [1–3], and has become widely practiced in recent years [4–6]. Its rapid spread may be attributed not only to its adoption by urologists but also by endosurgeons, and the procedure itself has become divided into two camps. General surgeons are overwhelmingly in favor of the anterior approach, while urologists seem to prefer the retroperitoneal approach [7]. The main technical problem with the transperitoneal anterior approach is whether a good view of the operating site can be obtained without damage to the solid organs, especially in right adrenalectomy. The procedure for the lateral approach, first described by Gagner [8], addressed this question. The liver in the right side and the spleen in the left side can be put outside the view unforcibly by their own weight, and by small additional time and effort could give the broad visual field needed to remove the adrenal gland safely. We first started laparoscopic adrenalectomy a year ago using the anterior approach, but have recently switched to the lateral approach.

The retroperitoneal foregut cyst is considered to have its origin in aberrantly migrated cells of the primitive ventral foregut which has multipotentiality. It can develop into a bronchogenic cyst which has definite cartilageous content, and can otherwise develop into an esophageal, gastric or enteric cyst which has distinctive features characteristic of each organ. The cystic tumor in this report was found to be lined by ciliated epithelium, but lack cartilageous component which is necessary for a diagnosis of bronchogenic cyst. So it could not be classified strictly, and was considered to be appropriately called 'undifferentiated foregut cyst', as described by Colby *et al.* [9]. It is rare for a foregut-origin cyst to be located in the retroperitoneal cavity and, in literature, only 19 cases have been reported up to now [10,11]. This is the second reported case treated endosurgically [12].

In this case, a retroperitoneal tumor was unsuspectedly resected laparoscopically as an adrenal tumor. Incidental adrenal non-functioning tumors are reported to contain 4% adrenal cancers [13], which are likely to be 6.5 cm or larger in diameter. On the other hand, retroperitoneal tumors, though including various entities, are reported to have 80% malignancy as a whole [14]. This means that endosurgical forceps can possibly damage the tumor capsule inadvertently, causing tumor cells to scatter or the liquid content leak out.

Thus, when treating paravertebral incidentaloma beneath the diaphragm, we should also take into consideration the possibility of retroperitoneal tumor unrelated to the adrenal gland. In cases in which retroperitoneal tumor is suspected prior to the operation, we would recommend that open surgery be chosen, since benignancy cannot be predicted.

### References

[1] Higashihara, E., Tanaka, Y., Horie, S. *et al.* Laparoscopic adrenalectomy: the initial 3 cases. *J. Urol.* 1993; **149**: 973–976.

[2] Suzuki, K., Kageyama, S., Ueda, D. *et al.* Laparoscopic adrenalectomy: clinical experience with 12 cases. *J. Urol.* 1993; **150**: 1099–1102.

[3] Matsuda, T., Terachi, T. and Yoshida, O. Laparoscopic adrenalectomy: the surgical technique and initial results of 13 cases. *Min. Invas. Ther.* 1993; **2**: 123–127.

[4] De Canniere, L., Michel, L., Hamoir, E. *et al.* Videoendoscopic adrenalectomy: multicentric study from the Belgian group for endoscopic surgery (BGES). *Int. Surg.* 1996; **81**: 6–8.

[5] Staren, E.D. and Prinz, R.A. Adrenalectomy in the era of laparoscopy. *Surgery* 1996; **120**: 706–709.

[6] Matsuda, T., Terachi, T. and Yoshida, O. Laparoscopy in urology: present status, controversies, and future directions. *Int. J. Urol.* 1996; **3**: 83–97.

[7] Gaur, D.D., Agarwal, D.K. and Purohit, K.C. Retroperitoneal laparoscopic nephrectomy: initial case report. *J. Urol.* 1993; **149**: 103–105.

[8] Gagner, M., Lacroix, A. and Bolte, E. Laparoscopic adrenalectomy in Cushing's syndrome and pheochromocytoma. *N. Engl. J. Med.* 1992; **327**: 1033.

[9] Colby, T.V., Koss, M.N. and Travis, W.D. Tumors of the lower respiratory tract. In: Rosai, J. and Sobin, L.H., eds. *Atlas of Tumor Pathology*, 3rd series, fascicle 13. Washington, DC: Armed Forces Institute of Pathology, 1995: pp. 16–20.

[10] Doggett, R.S., Carty, S.E. and Clarke, M.R. Retroperitoneal bronchogenic cyst masquerading clinically and radiologically as a phaeochromocytoma. *Virchows Archiv.* 1997; **431**: 73–76.

[11] Cetinkursun, S., Ozturk, H., Celasun, B. *et al.* Isolated abdominal bronchogenic cyst: a case report. *Eur. J. Pediatr. Surg.* 1997; **7**: 103–105.

[12] Tokuda, N., Naito, S., Uozumi, J. *et al.* A retroperitoneal bronchogenic cyst treated with laparoscopic surgery. *J. Urol.* 1997; **157**: 619.

[13] Aso, Y. and Homma, Y. A survey on incidental adrenal tumors in Japan. *J. Urol.* 1992; **147**: 1478–1481.

[14] Armstrong, J.R. and Cohn, I. Primary malignant retroperitoneal tumors. *Am. J. Surg.* 1965; **110**: 937–943.

